# Energy-Efficient Data Collection Using Autonomous Underwater Glider: A Reinforcement Learning Formulation

**DOI:** 10.3390/s20133758

**Published:** 2020-07-04

**Authors:** Xinbin Li, Xianglin Xu, Lei Yan, Haihong Zhao, Tongwei Zhang

**Affiliations:** 1 Institute of Electrical Engineering, Yanshan University, Qinhuangdao 066004, China; xxl6@outlook.com (X.X.); ysuyanl@163.com (L.Y.); 18713535112@163.com (H.Z.); 2 National Deep Sea Center, Qingdao 266237, China; tong-wei.zhang@outlook.com

**Keywords:** autonomous underwater glider, underwater data collection, multi-armed bandit, indexable threshold policy, energy efficiency

## Abstract

The autonomous underwater glider has attracted enormous interest for underwater activities, especially in long-term and large-scale underwater data collection. In this paper, we focus on the application of gliders gathering data from underwater sensor networks over underwater acoustic channels. However, this application suffers from a rapidly time-varying environment and limited energy. To optimize the performance of data collection and maximize the network lifetime, we propose a distributed, energy-efficient sensor scheduling algorithm based on the multi-armed bandit formulation. Besides, we design an indexable threshold policy to tradeoff between the data quality and the collection delay. Moreover, to reduce the computational complexity, we divide the proposed algorithm into off-line computation and on-line scheduling parts. Simulation results indicate that the proposed policy significantly improves the performance of the data collection and reduces the energy consumption. They prove the effectiveness of the threshold, which could reduce the collection delay by at least 10% while guaranteeing the data quality.

## 1. Introduction

The ocean is rich in biological and mineral resources, making the exploitation and usage of ocean resources receive more and more attention [1,2,3]. Ocean data are considered to be the basis of marine research, development, and utility, and the advancements in marine science and technology promote the development of ocean data collection research. Existing research on underwater data collection focused on two aspects: collecting data via multi-hop transmission, which has low latency and uneven energy consumption, or underwater vehicles, which have less transmission energy consumption and large delay [4]. Of these two aspects, the underwater vehicle is more suitable for practical applications, because energy efficiency is a significant objective in underwater data collection missions. Generally, autonomous underwater vehicles (AUVs) are applied in data collection missions because they are flexible and maneuverable, and they can adaptively gather data from underwater acoustic sensor networks (UASNs). However, AUVs are restricted to relatively short-term deployments (weeks) and small areas due to the huge consumption of movement.

Gliders are designed for long-range and long-duration missions. Different from AUVs, gliders are propelled by a buoyancy engine instead of a standard propeller. Thus, gliders are slow moving (about 0.6 knots) and low power and have low self-noise [5,6]; they are reusable and able to operate for several months while covering large distances (thousands of kilometers). These advantages make gliders ideal platforms for underwater data collection. Besides, many research works focused on the movement stability and path optimization of gliders [7,8]. In recent years, gliders have played an indispensable role in oceanography, for example the Sea-wing underwater glider participated in studying the influence of the wind on the cooling of the upper mixed layer in the northern South China Sea and measured temperature, salinity, and pressure [9], and the Petrel II glider was used to analyze the underwater noise spectrogram [10]. In those applications, gliders gathered ocean data via various sensors they carried. Nevertheless, studies have shown that sensors account for a great proportion of the total energy consumption [11], and the resulting on-board data storage places an enormous burden on the embedded computing units. Thus, we consider removing the environment sensing function and corresponding sensors that produce the necessary exploratory data, from gliders to UASNs, and establish a data collection system composed of gliders and UASNs.

There are many intractable problems during the application of gliders in UASNs. First, underwater communications rely on acoustic channel links, which are easily affected by multipath propagation and Doppler shift. The low propagation speed of the acoustic signal is another main limiting factor for efficient underwater communication [12] and causes the “outdated” feedback of the channel state information (CSI). Underwater sensor nodes are charged by batteries for long-term functioning, and batteries are difficult to recharge or replace [13] during the service period. A learning-based algorithm is effective against the limited energy and time-varying environment [14]. Finally, the hardware constraints of sensor nodes such as various CPUs’ capability, limited memory capacities [15], and the large data they have to store and process pose challenges to the collection delay and computational overhead of underwater data collection to avoid over-utilization of sensor nodes.

The application of gliders to collecting underwater data from UASNs is poorly researched. Since the AUV is a thoroughly studied agent in data collection missions with UASNs, the data collection algorithm of gliders could learn from the case for AUVs. AUVs are propelled by active propulsion, and they can adaptively adjust their travel path according to the algorithm results for better data collection performance [4,16,17]. However, such path planning algorithms do not apply to gliders, because gliders move up or down and travel along a saw-tooth trajectory, that is, if the glider intends to arrive at a node at the same level, it must move up, then down (or down, then up). In [4], UASNs were divided into two layers and different algorithms were employed: in the lower layer was a modified path planning scheme; while in the upper layer, the AUV dove from top to bottom and collected the data from gateway nodes, which received data packages from other nodes based on a multi-hop algorithm, and in this layer, the energy consumption increased with the diving path increase. In addition, a proper routing protocol is conducive to the good performance of a multi-hop algorithm [18]. To meet the challenges of the underwater environment, the Q learning algorithm of reinforcement learning has been widely used to optimize the system performance [19,20]. Reinforcement learning can learn on-line from the environment without prior knowledge of the input and output, and then, it is always applied underwater to optimize the performance and achieve the objectives. In addition, in [21], a stationary multi-armed bandit (MAB) formalism was applied for maximizing underwater acoustic data transmission through adaptive positioning of a mobile relay.

The aforementioned problems pose two challenges to the system: collecting underwater data given the special movement characteristics of the glider and optimizing the data collection performance under the fast-changing environment. Different from algorithms for AUVs, we propose a sensor scheduling algorithm for gliders and design an indexable threshold policy to optimize the data collection performance. Since the underwater environment changes over time and sensor nodes are independent of each other, we model the scheduling problem as a multi-armed bandit problem and solve it using a modified Gittins index policy. The basic idea is to set up a threshold for the index, and when the index falls below the threshold, the current selected sensor node is switched; otherwise, it is not. To reduce the computational overhead, we divide the process into off-line and on-line parts. The simulation results show that the data collection performance of our proposed policy outperforms traditional schemes and illustrate the effectiveness of the threshold on the collection delay.

The rest of this paper is organized as follows. In Section 2, we present the necessary models and the optimization objectives. A reinforcement learning formulation is given in Section 3. Section 4 discusses the sensor scheduling process and the effect of the proposed threshold. Simulation results are provided in Section 5. Finally, we conclude the paper in Section 6.

## 2. Preliminaries

### 2.1. Principle of Autonomous Underwater Gliders

**Figure 1:**
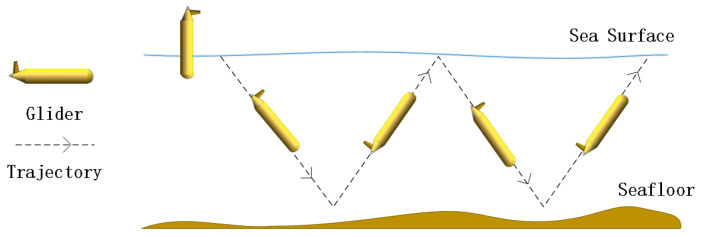
The saw-tooth trajectory of a glider.

The underwater glider is an emerging underwater vehicle aimed at long-term and long-range underwater missions. The driving force of gliders is induced by changing the buoyancy through controlling the flow of hydraulic oil into or out of the external oil bladder and achieves vertical motion including upwards and downwards. Gliders are steered by the buoyancy-regulating device that adjusts the trajectory in the vertical direction, but also the rudder mitigating deviation in the horizontal direction [22,23]. Thus, gliders move underwater up and down and repeat this movement, and they travel along a saw-tooth trajectory, as shown in Figure 1.

The lack of a propeller system makes gliders energy efficient and acoustically quiet, which also makes gliders sensitive to the ocean current. The gliding range of a glider is affected by its initial angle, gliding depth, speed, and the energy consumption of each subsystem onboard [11]. Underwater gliders are reusable and can be constructed quickly. Thus, the virtues of the underwater glider make it highly suitable for underwater acoustic data collection.

### 2.2. System Model

**Figure 2:**
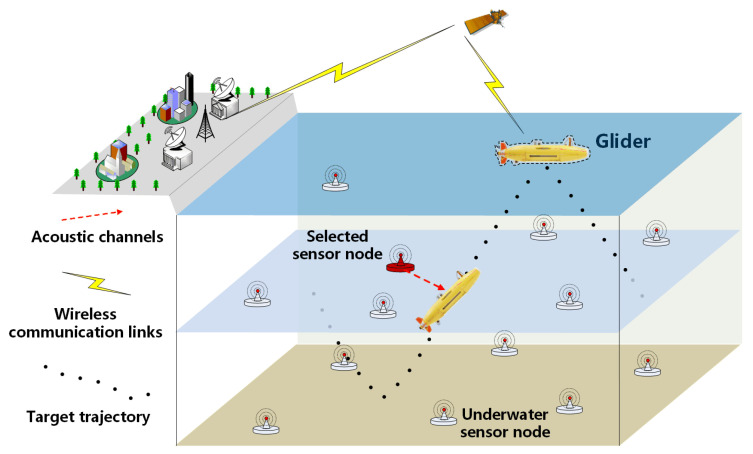
System model composed of one glider and UASNs.

We considered a distributed underwater data collection system where the glider worked as the data collection agent and continuously gathered data information from the UASN, which included multiple sensor nodes. The three-dimensional system model [24] is shown in Figure 2. While the glider moved underwater along the saw-tooth trajectory, it always collected data from the sensor nodes chosen by the embedded data collection algorithm of the glider; note that only one senor node could be chosen at a time. While the glider moved at the sea surface, it transmitted the data to the base station via satellite communication [25]. On the other hand, all sensor nodes were static and anchored to the seafloor by cables, floating at different heights from the seabed. Each node was equipped with multiple sensors (such as seismic sensors) and an acoustic modem for sensing and transmitting. When a glider moved closer, sensor nodes in the glider’s communication range would wake up, sense the environment, and transmit sensor data to the glider using the acoustic signals if chosen; whereas, sensor nodes knew nothing about the states of any others. Powered by batteries, sensor nodes could not be recharged during their service time. Once the energy ran out, the node was dead.

### 2.3. Acoustic Channel Model

Acoustic channels change rapidly and suffer from limited bandwidth, large delay spread, multi-path propagation, and so on. The channel fading mainly changes with distance and frequency; thus, we modeled acoustic channels in two aspects including large-scale fading and small-scale fading [26] according to the scope of the channel variations, the former involving plenty of wavelengths and the latter a few wavelengths.

Time was divided into *T* time slots, and we used t∈{1,…,T} to denote any arbitrary time slot. The acoustic channel link state was modeled by a finite-state Markov chain [27], and we assumed that the channel link state remained unchanged during each time slot and was allowed to change at each time instant in a Markovian manner. The channel state of node *i* at time *t* can be expressed as:

(1) $$B_{t}^{i}=\left(\zeta_{t}^{i},\delta_{t}^{i}\right),$$

where $\zeta_{t}^{i}$ represents the current state of the small-scale fading of node *i* at time *t* and $l_{t}^{i}$ stands for the state of large-scale fading.

Small-scale fading includes scattering and Doppler shifting, which has a finite state space denoted by $C=\left\{c_{1},\ldots ,c_{L}\right\}$. The state of small-scale fading $\zeta^{i}$ evolves according to the special Markov transition probability matrix of each node. Let $\varphi^{i}\left(c_{v},c_{u}\right)$ denote the probability of sensor node *i* when the state $\zeta^{i}$ moves from state $c_{v}$ to state $c_{u}$ at time *t*:

(2) $$\varphi^{i}\left(c_{v},c_{u}\right)=P\left(\zeta_{t+1}^{i}=c_{u}|\zeta_{t}^{i}=c_{v}\right),$$

where $c_{v},c_{u}\in C^{i}$ and v,u=1,…,L. Then, the corresponding L×L transition probability matrix is defined as:

(3) $$\Psi^{i}={\left[\varphi^{i}\left(c_{v},c_{u}\right)\right]}_{L\times L}.$$

Large-scale fading is caused by the location uncertainty and environmental changes [28], and it mainly appears as transmission loss and absorption loss during the sound propagation, large-scale fading is a function of distance *l* and frequency *f*,

(4) LS(l,f)=n×10logl+α(f)l,

where *n* is a scaling constant, *l* is a variable that denotes the distance between the transmitter and receiver, and α is the absorption coefficient, which is a function of frequency *f* and can be obtained using the Thorp experience formula:    

(5) $$\begin{array}{c} \alpha \left(f\right)=0.11\frac{f^{2}}{1+f^{2}}+44\frac{f^{2}}{4100+f^{2}}+3.0\times 10^{-4}f^{2}\\ +3.3\times 10^{-3}\mathrm{dB}/\mathrm{km}. \end{array}$$

In this paper, we assumed all acoustic modems worked at the same frequency, which made the distance deterministic. To measure the influence of large-scale fading on the quality of collected data properly, the continuous distance state between the chosen node and the glider was divided into *K* levels and evolved in a *K*-state Markov chain. The state profile was $D=\left\{d_{1},\cdots ,d_{K}\right\}$. Then, let $\phi^{i}\left(d_{f},d_{g}\right)$ represent the probability that $\delta^{i}$ moves from state $d_{v}$ to state $d_{u}$ at time *t*, which is defined as:

(6) $$\phi^{i}\left(d_{f},d_{g}\right)=P\left(\delta_{t+1}^{i}=d_{g}|\delta_{t}^{i}=d_{f}\right),$$

where $d_{f}$, $d_{g}$, and f,g=1,…,K. The K×K transition probability matrix of large-scale fading is defined as:

(7) $$\Phi_{i}={\left[\phi^{i}\left(d_{f},d_{g}\right)\right]}_{K\times K}.$$

### 2.4. Energy Model

Underwater sensor nodes are powered by disposable batteries, with limited energy, and they are able to detect their residual energy locally and exactly. For simplicity, we divided the continuous residual energy state into *H* levels, denoted by $E=\left\{\varepsilon_{1},\ldots ,\varepsilon_{H}\right\}$. Let variable $e^{i}$ denote the residual energy state of sensor node *i* and then $e^{i}\in E$. The residual energy state $e^{i}$ evolves likewise in a finite-state Markov chain according to a transition probability matrix, denoted by:

(8) $$\Theta^{i}={\left[\theta^{i}\left(\varepsilon_{k},\varepsilon_{j}\right)\right]}_{H\times H},$$

where $\theta^{i}\left(\varepsilon_{k},\varepsilon_{j}\right)$ represents the probability the residual energy state $e^{i}$ of sensor node *i* evolves from state $\varepsilon_{k}$ to state $\varepsilon_{j}$ at time *t* and is defined as:

(9) $$\theta^{i}\left(\varepsilon_{k},\varepsilon_{j}\right)=P\left(e_{t+1}^{i}=\varepsilon_{j}|e_{t}^{i}=\varepsilon_{k}\right),$$

where $\varepsilon_{k},\varepsilon_{j}\in E$, and k,j=1,…,H.

Apart from the residual energy, we modeled the energy consumption required for a successful transmission of sensor data, so that sensor nodes could choose the lowest transmission power that met the energy requirement for saving the system energy and maximizing the network lifetime. We use γ to denote the minimum transmission energy required for successful transmission. γ is determined by the current channel state $B^{i}$, and the better the channel state is, the less the transmission energy required  [29]. Assume *W* denotes the energy consumed in transmission, and it has *L* choices: $W\in \left\{w_{1},\ldots ,w_{L}\right\}$ and $0<w_{1}<\cdots <w_{L}<\infty$. The distribution of *W* is determined by the channel distribution. The relationship between the minimum transmission energy γ and the channel states is defined as:

(10) $$\gamma =\frac{\bar{E}}{B},$$

where $\bar{E}$ is the energy consumption for a successful transmission when the channel gain is one. Therefore, the energy consumption *W* can be defined as follows:

(11) $$W=\left\{\begin{array}{cc} \min_{k}\left\{w_{k}:w_{k}\geq \gamma \right\}, & \;\mathrm{if}\;B\geq \frac{\bar{E}}{w_{L}}\\ \infty , & \;\mathrm{otherwise}\; \end{array}\right.$$

When the channel link experiences deep fading and the energy required for a successful transmission exceeds the residual energy, that is $B<\frac{\bar{E}}{w_{L}}$, the sensor node fails in this data transmission, and we use *∞* to represent this state.

### 2.5. Objectives

In this paper, we established a data collection system composed of a glider and UASNs and modeled the acoustic channel and the energy. The optimization objectives were as follows:

- Optimize the data collection performance of the sensor scheduling algorithm. The prime objective of the data collection mission was to guarantee the total quality of the collected data. A better acoustic channel leads to a more reliable transmission with a lower bit error rate (BER); however, the time-varying environment puts forward a huge challenge.
- Maximize the network lifetime. Since underwater sensor nodes are charged by batteries, it is essential to use energy efficiently. To minimize the energy consumption is to maximize the network lifetime.
- Minimize the computation overhead. The computing power and storage capacity of the glider and sensor nodes were limited, which called for a simple algorithm with less computation and communication cost.

## 3. Problem Formulation

In this paper, we considered an underwater data collection system composed of one glider and UASNs. Since the glider lacked active propulsion and moved along a saw-tooth trajectory, we proposed a sensor scheduling algorithm for the glider to gather sensor data from UASNs. Typically, the glider, as the data collection agent, always faced an exploration vs. exploitation dilemma whether to try unknown nodes with a potential for better performance or continue with known nodes to ensure quality. We formulated this dilemma as a multi-armed bandit (MAB) problem, an important method of reinforcement learning, and solved this problem using an indexable policy.

### 3.1. System State and Reward of The Multi-Armed Bandit Problem

The MAB problem has been widely studied in the context of the finite-horizon scheduling problem. In our proposed data collection system, we regarded the *N* sensor nodes as “arms” and the glider as the “player”, and then, we formulated the sensor scheduling problem by dynamically choosing one executive node among *N* candidates at each decision time, as a stochastic MAB system. In this system, each arm had a finite state space X and a corresponding transition probability matrix P. At each decision time t=0,1,2,…, each “arm” was in a state $x_{i}^{t}\in X$. According to the states and the observation history of all nodes, only one node (arm) was selected to be active, and others remained frozen. When selected, the sensor node provided a reward $r^{i}\left(x^{i}\right)$, which was associated with the environment and reflected it, and then, the state of the sensor node evolved in a Markov manner with the transition probability $p^{i}$, which is an element of matrix P.

Therefore, each sensor node *i*, i=1,…,N in the stochastic MAB problem was characterized by a triple $\left(X,r^{i}\left(\cdot \right),\rho^{i}\right)$, where X is the finite state space assumed to be identical for all nodes; $r^{i}\left(\cdot \right)=r^{i}\left(x^{i}\right)\left(x^{i}\in X\right)$ is a state-dependent reward function of sensor node *i*, and $\rho^{i}=\rho^{i}\left(x^{i},j\right)\left(x^{i},j\in X\right)$ is the corresponding transition probability function over state space X.

As modeled in Section 2, the state of a sensor node is the combination of the residual energy state and the channel state, which includes large-scale fading and small-state fading. Accordingly, the state space X is made up of these three parts, defined as:

(12) X=[C,D,E].

where C represents the state space of small-scale fading, D represents the state space of large-scale fading, and E is the state space of residual energy.

Note that the state $x_{t}^{i}$ evolves over the state space X in a Markovian fashion, and the evolvement of each of the three components is independent of the others. Assume the current state is $x_{t}^{i}=m$, then the transition probability $p^{i}$ when the state jumps from *m* to *n* is defined as:    

(13) $$p^{i}\left(m,n\right)=P\left(x_{t+1}^{i}=n|x_{t}^{i}=m\right).$$

where m,n∈X. $p^{i}$ is the element of P. The transition probability matrix P of each sensor node *i* is the Cartesian product of the transition probability matrices of large-scale fading, small-scale fading, and the residual energy, and it is defined as:

(14) $$P={\left[\varphi^{i}\left(c_{v},c_{u}\right),\phi^{i}\left(\gamma_{f},\gamma_{g}\right),\theta^{i}\left(\varepsilon_{k},\varepsilon_{j}\right)\right]}_{G\times G},$$

where $\varphi^{i}\left(c_{v},c_{u}\right),\phi^{i}\left(\gamma_{f},\gamma_{g}\right)$ and $\theta^{i}\left(\varepsilon_{k},\varepsilon_{j}\right)$ are defined in Section 2 and G=L×K×H.

In the stochastic MAB problem, the system reward embodies the objectives and reflects the environment. To minimize the BER and maximize the network lifetime, the system reward is designed to be a function of channel quality and energy consumption [30]. Therefore, the system reward is defined as:

(15) $$r^{i}=R\left(\omega_{B}B\left(\zeta^{i},\delta^{i}\right),\omega_{W}W^{i}\times B^{i},\omega_{E}E\left(e^{i}\right)\right),$$

where $\omega_{B},\omega_{W}$ and $\omega_{E}$ are coefficients taking the value of 0, 1, or −1. B(·) is the BER function of the large-scale fading and small-scale fading of acoustic channels; E(·) is the residual energy function; and $W^{i}$ is the energy consumption variable depending on the channel state. The above-mentioned parameters are listed in Table 1.

### 3.2. The Gittins Index

In this stochastic MAB framework, we assumed at each time slot that only one arm could be operated; each arm was allowed to evolve in the Markov manner when selected to be active; otherwise, it remained frozen, and the arms were independent of each other. Based on the above hypotheses, a feasible solution to the MAB problem was to set it up as a Markov decision process (MDP) and solve it using Markov decision theory. However, this solution resulted huge computational complexity with the increase of the bandit processes. Gittins [31] developed an index policy, called the Gittins index policy, for the MAB problem to break the curse of dimensionality and concluded that instead of solving the *n*-dimensional MDP, an optimal solution of the MAB problem could be obtained by solving *n* 1-dimensional optimization problems. The details of the mathematical formulation of this index policy are as follows.

At each time slot t=1,…,T, the “player” will select one of *N* available nodes to be active. Hence, each sensor node could be described as two states: either played or not, which can be described by a Bernoulli trial:

(16) $$u_{t}^{i}=\left\{\begin{array}{cc} 1, & \;\mathrm{if}\;\mathrm{arm}\;i\;\mathrm{is}\;\mathrm{chosen}\;\mathrm{to}\;\mathrm{be}\;\mathrm{active}\;\\ 0, & \;\mathrm{otherwise}\; \end{array}\right.$$

where $u_{t}=\left(u_{t}^{1},\ldots ,u_{t}^{N}\right)$ denote the working states of all arms at time *t*. At each time slot, $u_{t}$ has only one nonzero component, and thus, we have:

(17) $$\sum_{i=1}^{N}u_{t}^{i}=1,\forall t.$$

If selected, the state of the sensor node evolves in the Markov manner; otherwise, the state remains frozen. The state of all sensor nodes changes by the following rule:

(18) $$x_{t+1}^{i}=\left\{\begin{array}{cc} M^{i}\left(x_{t}^{i},p^{i}\right), & \;\mathrm{if}\;u_{t}^{i}=1\\ x_{t}^{i}, & \;\mathrm{if}\;u_{t}^{i}=0 \end{array}\right.$$

where $M^{i}\left(\cdot \right),i=1,\ldots ,N$ is a Markov chain function depending on the current state $x_{t}^{i}$ of arm *i* and the corresponding transition probability $p^{i}$. Based on the problem formulation of MAB, we aimed to maximize the expected total discounted reward *V* after discounting all rewards to Time 0 with the discount factor β. The expected total discounted reward is defined as:

(19) $$V\left(x_{0}\right)=E_{\pi }\left\{\sum_{t=0}^{\infty }\sum_{i=1}^{N}\beta^{t}\left(r^{i}\left(x_{t}^{i}\right)u_{t}^{i}\right)|x_{0}=x_{0}\right\}.$$

where π is the decision strategy that maximize the expected total discounted reward; $x_{0}=\left(x_{0}^{1},\ldots ,x_{0}^{N}\right)$ is the given initial states of all sensor nodes; β∈(0,1) is the discount factor, which indicates the relative importance of future returns to current returns. When β is close to one, this leads to a “far-sighted” evaluation, and when β is close to zero, this leads to a “myopic” evaluation.

For each sensor node *i* and its corresponding state $x^{i}$, the Gittins index $v^{i}$ can be calculated as a function of its state $x^{i}$ that maximize the expected discounted reward per unit of expected discounted time [32], which is defined as follows:

(20) $$v^{i}\left(x^{i}\right)=\max_{\tau >0}\frac{E\left\{\sum_{t=0}^{\tau }\beta^{t}r^{i}\left(x_{t}^{i}\right)|x_{0}^{i}=x^{i}\right\}}{E\left\{\sum_{t=0}^{\tau }\beta^{t}|x_{0}^{i}=x^{i}\right\}}$$

where τ is the stopping times defined on ${\left\{\sigma \left(x_{1}^{i},\ldots ,x_{t}^{i}\right)\right\}}_{t=1}^{\infty }$. As characterized by Gittins and Jone [33], the optimal strategy is to select the arm with the highest Gittins index at each time slot. The Gittins index could be computed off-line or on-line. For a finite state space, the off-line implementation is simpler and more convenient compared with the on-line implementation, which is suitable for our problem formulation.

## 4. Sensor Scheduling

In this paper, we designed a data collection system in which the glider gathered sensor data from a UASN instead of carried sensors. Based on the special movement characteristics of the glider, we addressed a sensor scheduling algorithm, which was formulated as a stochastic MAB problem solved by an index policy. In this section, we propose an indexable threshold policy for optimally scheduling sensors and achieving our objectives.

### 4.1. Scheduling Process

A distributed sensor scheduling algorithm was proposed for minimizing the BER of collected data and maximizing the network lifetime. Sensor nodes within the communication range of the glider were considered as “arms” of the MAB framework. To reduce the computational burden of the glider and sensor nodes, we divided the whole data collection process into two parts: off-line computation and on-line scheduling. In the off-line parts, the glider calculated the indices of all possible states for each sensor node and saved them in an index table; while in the on-line process, the indexes of sensor nodes were updated in real time, and sensor nodes were scheduled based on current indexes, which could be conveniently gained from the index table according to the detected channel and energy state, instead of calculating the index each time. Initially, sensor nodes stayed asleep, and they were wakened by the glider when they received the handshaking requests from it. During the handshaking process, sensor nodes and the glider exchanged the policy information, the index table, and the current state indexes of sensor nodes. After the handshaking, the scheduling process began. At each time slot, the glider would select one sensor node according to the sensor scheduling algorithm to gather its sensor data; if at the next time slot, the glider switched to another sensor node, a “broadcast process” in which the current node broadcast its index information throughout the whole network to inform other nodes to transmit their current state indices to the glider occurred.

Typically, the optimal solution to the MAB problem for scheduling sensor nodes was proposed by Gittins as choosing the arm with the highest Gittins index at each time. However, the fast-changing underwater environment leads to frequent “broadcast processes”, which consume much energy and shorten the network lifetime. Thus, to reduce the frequency of the “broadcast processes” and save the system energy, we proposed a threshold policy based on the Gittins index policy. A threshold T was set up on the Gittins index policy, which split the whole scheduling process into a set of epochs: during each epoch, the sensor node selected at the beginning of this epoch would continue to be selected until its index fell to the threshold; before the next epoch, the glider chose a sensor node with the highest index based on its knowledge of all sensor nodes for the successive epoch. Therefore, the selection rule of our proposed indexable threshold policy π for scheduling sensor nodes is defined as:

(21) $$i_{t+1}=\left\{\begin{array}{cc} \underset{i=1,\ldots ,N}{\mathrm{argmax}}\left\{v^{i}\left(x_{t}^{i}\right)\right\}, & \;\mathrm{if}\;v^{i}\left(x_{t}^{i}\right)<T\\ i_{t}, & \;\mathrm{if}\;v^{i}\left(x_{t}^{i}\right)\geq T \end{array}\right.$$

where $i_{t}$ represents the selected arm at time *t*.

Note that when energy ran out, the sensor node was dead and no longer engaged in the data collection process, then there was nothing returned from it. This situation was seen as returning zero, which was less than the threshold. The details of our proposed indexable threshold policy for sensor scheduling are summarized in Algorithm 1.

### 4.2. Near Optimality

The sensor scheduling process was divided into two phases due to the threshold T:

The first phase: There existed at least one arm with indexes exceeding the threshold T. In this phase, once an arm i,i∈{1,…,N} was chosen, it continued to be active until its index decreased to the threshold, and then, another arm was to be active. The process of this phase could be converted into one special arm-acquiring problem with simple interchanges: first, we introduced a total order ⪰ on all arms based on the index of the initial state of each arm, which is given by:

(22) $$\forall i,j\in \left\{1,\ldots ,N\right\},i⪰j\Leftrightarrow v^{i}\left(x_{0}^{i}\right)⩾v^{j}\left(x_{0}^{j}\right).$$

Thus, we denoted arms by $\alpha_{1},\ldots ,\alpha_{N}$ according to the total order $\alpha_{1}⪰\alpha_{2}⪰\cdots ⪰\alpha_{N}$. We assumed there was a fictitious arm $\alpha_{0}$ with fixed state and index, which was equivalent to the threshold T. Arms arrived in decreasing order. At the beginning of this phase, only arm $\alpha_{1}$ and the fictitious arm $\alpha_{0}$ were available. Since the threshold was the lower bounds of the index in this phase, arm $\alpha_{1}$ was chosen at each time slot before the next arm arrived due to the index rule of always choosing the arm with the highest index. Then, the next arm $\alpha_{2}$ arrived at the time slot when the index of arm $\alpha_{1}$ failed to be greater than that of $\alpha_{0}$. New arms arrived as time went on until the last arm $\alpha_{N}$ arrived, and then, the first phase terminated when the index of arm $\alpha_{N}$ became less than that of $\alpha_{0}$. The above special arm-acquiring problem could be solved by classical Gittins index policy for an optimal solution; however, the irrevocable property of decisions in our situation made this process near-optimal [32] and sometimes optimal.
**Algorithm 1** The proposed indexable threshold policy.**Input:** transition probability matrix P; discounted factor β; reward vector r; initial state vector $x_{0}$; horizon length *T*; threshold T;**Output:** the sensor scheduling sequence of the policy π;  1: *Off-line:*  2: **for**
i=1,…,N
**do**  3:  Calculate the Gittins indices $v^{i}$ of all states for each sensor node based on (20) and save them in the index table;  4: **end for**  5: Create the index vector $g=v^{i}\left(x_{0}\right)$;  6: Find the arm with maximum Gittins index, and save it as the initial active arm $i_{0}$.  7: *On-line:*  8: **for**
t=1,2,⋯,T
**do**  9:  $x_{t}^{i_{t}}=M^{i_{t-1}}\left(x_{t-1}^{i_{t-1}},\rho^{i_{t-1}}\right)$  10:  Search the index table and acquire the current index $v^{i_{t}}$  11:  Update the index vector g  12:  Compare $v^{i_{t}}$ with the threshold T  13:  **if**
$v^{i_{t}}<T$ or *arm*
$i_{t}$
*dies*
**then**  14:   arm $i_{t}$ broadcasts its index information throughout the network  15:   $i_{t+1}=\underset{i=1,\ldots ,N}{\mathrm{argmax}}\left\{v^{i}\left(x_{t}^{i}\right)\right\}$  16:  **else**  17:   $i_{t+1}=i_{t}$  18:  **end if**  19: **end for**

The second phase: There existed no arm with indexes exceeding threshold T. In the second phase, since the threshold T was greater than all arms and the state of arms was unlikely to evolve to a good one with an index much higher than T, the threshold had little effect on the decision tendency, compared to the classical Gittins index policy. Hence, the policy was optimal in the second part.

Above all, for the whole data collection process, our proposed indexable threshold policy was near-optimal, and sometimes optimal.

### 4.3. The Communication Cost

The communication cost was greatly reduced compared with the classical Gittins index policy due to the threshold. In the scheduling process, the communication cost was substantially reduced in the first phase and was almost equal to the classical Gittins index policy in the second phase because of the reduction of the “broadcast process” frequency in the first phase, which was addressed as follows.

Each arm could broadcast index information throughout the whole network once in the first phase. There were N−1 inactive sensor nodes and one glider; and the index information needed to transmit to *N* objects, and we assumed there were $\tilde{N}$ sensor nodes initialized with an index higher than the threshold, $\tilde{N}\leq N$. Hence, the total communication cost was $O(N\times \tilde{N})$. However, for the classical Gittins index policy without threshold, we assumed that the *N* arms operated $\left\{t_{1},\ldots ,t_{N}\right\}$ time slots respectively in the first phase, and the “broadcast process” happened at each time slot, then the total number of broadcasts was the sum the operation times of all arms, denoted by $\sum_{i=1}^{N}t_{i}$. We used $\bar{t}$ to represent the average operation times of arms, and the total communication cost was $O(\bar{t}\times N\times \tilde{N})$, obviously, $O\left(\bar{t}\times N\times \tilde{N}\right)\gg O\left(N\times \tilde{N}\right)$. Moreover, the communication cost gap grew with the average operation time $\bar{t}$. Further, the above discussion is proven with simulations in the next section.

## 5. Simulation and Performance Evaluation

In this section, we illuminate the effectiveness of our proposed sensor scheduling algorithm by simulations. First, we compare the proposed policy with the memoryless scheme and the random selection scheme to test the performance of the whole system. Then, we present the simulation results to demonstrate the effects of the threshold.

For simplicity, we assumed the state of the small-scale fading of acoustic channels could be weak (s1) or strong (s0), the state of the large-scale fading could be weak (d2), medium (d1), or strong (d0), and the state of residual energy could be high (e2), low (e1), or dead (e0). Hence, for each available sensor node, there were 18 states in the state space: s1d2e2, s1d2e1, s1d2e0, s1d1e2, s1d1e1, s1d1e0, s1d0e2, s1d0e1, s1d0e0, s0d2e2, s0d2e1, s0d2e0, s0d1e2, s0d1e1, s0d1e0, s0d0e2, s0d0e1, s0d0e0. For the small-scale fading, we set the transition probability of staying in the same state to be 0.8 and evolving to the other state to be 0.2. Then, for the large-scale fading, we set the transition probability of evolving to an adjacent state as 0.2 and the probability of jumping to a nonadjacent state as 0.1. Thus, the state transition probability matrices of the small-scale fading and the large-scale fading are:

(23) $$\Psi =\left[\begin{array}{cc} 0.8 & 0.2\\ 0.2 & 0.8 \end{array}\right]$$

and:

(24) $$\Phi =\left[\begin{array}{ccc} 0.7 & 0.2 & 0.1\\ 0.2 & 0.6 & 0.2\\ 0.1 & 0.2 & 0.7 \end{array}\right].$$

The residual energy model was a special case of Markov model, because the energy reduced successively and the batteries could not be recharged. Hence, we set the transition probability from a lower energy state to a higher state as zero, as well as from the high energy (e2) state to the dead (e0) state. Hence, the transition probability matrix of residual energy is:

(25) $$\Theta =\left[\begin{array}{ccc} 0.9 & 0.1 & 0\\ 0 & 0.9 & 0.1\\ 0 & 0 & 1 \end{array}\right].$$

Initially, the starting states of sensor nodes were randomly set with an assumption that all nodes were fully charged at the beginning of the simulation. We set the discount factor β=0.9 for the simulations.

The threshold T was set up on the index, and we define it as follows:

(26) T=⌈δ·U⌉,

where δ is the proportion to the maximum index value U. In the following simulations, we used δ to represent the threshold T. The detailed simulation parameters are listed in Table 2.

### 5.1. BER Performance

In this section, we study the BER performance of underwater data collection using the proposed policy. As we defined in (15), the reward was composed of three parts: the BER function, the energy consumption, and the residual energy function. To make the presentation clear, we only focused on the BER function of large-scale fading and small-scale fading on the acoustic channel; thus, we set the weights $\omega_{W}$ and $\omega_{E}$ to be zero and $\omega_{B}=1$, respectively. Without considering the energy, each sensor node had six states: s1d2, s1d1, s1d0, s0d2, s0d1, s0d0, and the corresponding state transition probability matrix could be easily acquired via calculating the Cartesian product of Ψ and Φ. We simulated the system with the same initial states for 100 time slots with N=50 available sensor nodes. For better analysis of the BER performance under an environment full of uncertainty, we took the average of the BER results during 100 time slots as the “average BER performance”, then we repeated the simulations 500 times. Besides, the threshold for this simulation was set to be σ=0.6.

**Figure 3:**
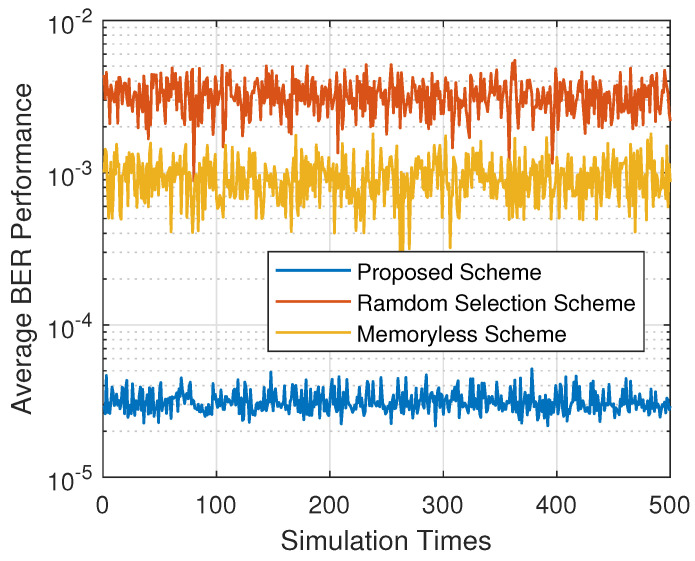
Average BER performance of different schemes.

Figure 3 shows the average BER performance of the proposed policy, the memoryless policy, and the random selection scheme. The memoryless scheme always selected the sensor node with the highest reward in the last time slot as the active one in the current time slot, and the random selection scheme randomly selected a node in every time slot. It can be perceived from Figure 3 that the average BER of our proposed policy was about $3\times 10^{-5}$, which was superior to the memoryless scheme (about $10^{-3}$) and the random selection scheme (about $4\times 10^{-3}$). This result confirmed the effectiveness of reinforcement learning, which learns the environment and predicts the environment. As for the memoryless scheme, the implied out-of-date state information caused poor performance.

**Figure 4:**
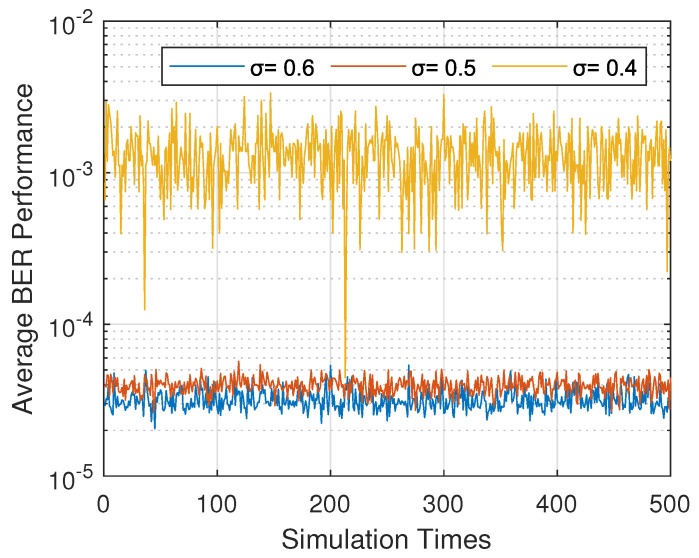
Average BER performance with different thresholds.

The average BER performance of our proposed policy varied with the threshold T. In Figure 4, we set σ=0.6,0.5, and 0.4 to present the effect of the threshold on the BER performance distinctly. It can be seen that when the threshold was set to σ=0.5, the corresponding BER performance was worse than the BER when σ=0.6 with a small gap. However, the performance gap became huge when the threshold was set to be σ=0.4. We do not show the BER performance with σ>0.6 here because it was only slightly better than when σ=0.6, and when σ<0.4, the great BER performance gap with a good one made it worthless. Therefore, the threshold had a limited effect on the BER performance of the data collection as long as σ≥0.5.

### 5.2. Network Lifetime

In this section, we pay attention to the system energy and study the network lifetime of different policies. To concentrate more on the system energy, we do not consider the reward of the acoustic channel here, that is, we set the reward weight $\omega_{B}=0$. As defined in Section 2, the energy consumption was related to the acoustic channel, and generally, the better the channel was, the less transmission energy required [29]. Therefore, each sensor node had 18 possible states, made up of the residual energy state and the energy consumption state. The state transition probability matrix was acquired by calculating the Cartesian product of Ψ and Θ. Simulations lasted 3000 times with 50 available sensor nodes at the beginning, and the discount factor was 0.9. Every sensor node was initialized with high energy (e2), and the number of alive nodes would decrease with time.

**Figure 5:**
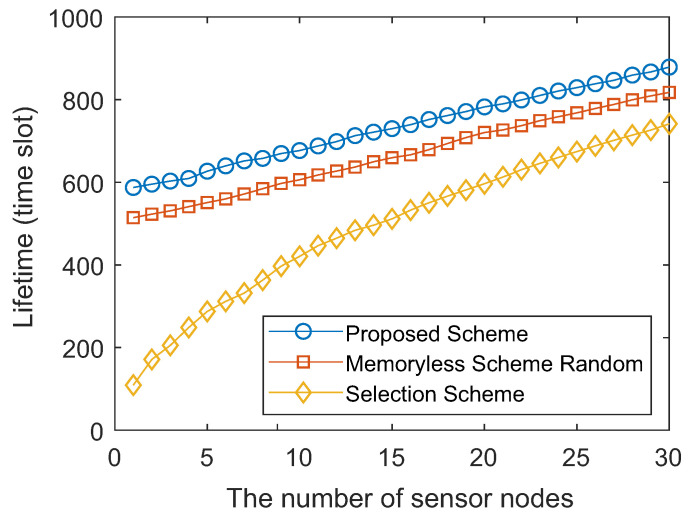
Network lifetime comparison of different schemes with different numbers of sensor nodes.

The network lifetime was defined as the number of data collections until the number of dead nodes reached the upper bound. Figure 5 compares different schemes and shows the changes in the network lifetime with varying upper bounds. The memoryless scheme always selected the node with the most residual energy, and the random selection scheme randomly selected one sensor node from those alive. It can be seen from Figure 5 that the network lifetime of all schemes increased with the growth of the dead sensor nodes. The first sensor node died at about the 600 th time slot in our proposed policy, whereas in the memoryless scheme and the random selection scheme, the first dead node appeared at about the 500 th time slot and the 100 th time slot, which suggested that our proposed policy performed better than the memoryless scheme and random selection scheme in energy efficiency.

### 5.3. The Effects of the Threshold

In this section, we focus on the effects of the threshold on the total reward and the collection delay, by comparing our proposed indexable threshold policy with varying thresholds: σ=1, σ=0.8, σ=0.6, and σ=0.4. Notably, when σ=1, the proposed policy worked in the same way as the canonical Gittins index policy (the canonical Gittins index policy). Typically, each sensor node had 18 states and was initialized with high energy (e2). The simulation ran for 100 time slots.

**Figure 6:**
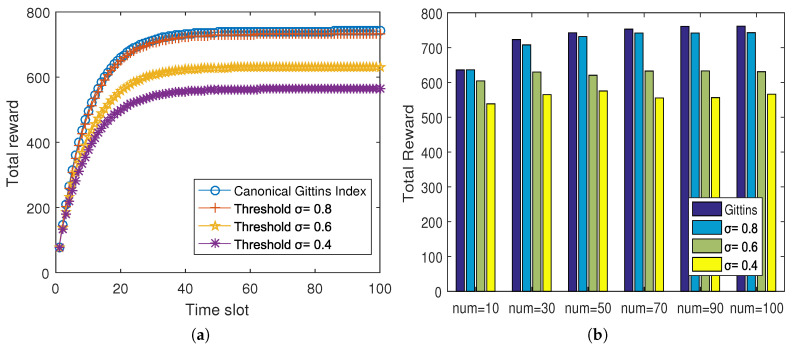
Total reward comparison with different thresholds. (**a**): total reward changes along with time slots; (**b**): total reward with different available sensor nodes.

**Figure 7:**
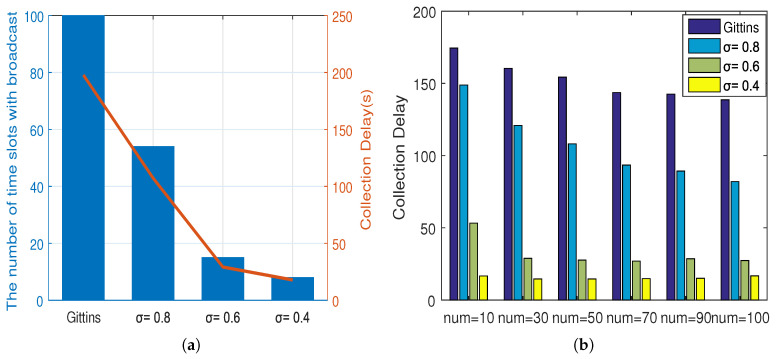
Collection delay comparison with different thresholds. (**a**): broadcast frequency and collection delay change according to the threshold; (**b**) collection delay with different available sensor nodes.

Figure 6 shows the expected total discounted rewards of the proposed policy with different thresholds. Figure 7 presents the broadcasting frequency and the collection delay with different thresholds. Since the index was acquired by searching the index table with little time consumption, the collection delay was mainly due to “broadcast process” frequency. As shown in Figure 6a and Figure 7a, when σ=0.8, the total reward was reduced by less than 2% compared with the canonical Gittins index policy, but the number of broadcast processes was reduced by about 40%. Likewise, when we set σ=0.6, our proposed scheme suffered about 15% of the total rewards, while the broadcasting frequency was reduced by 85%; and when σ=0.4, the total reward was cut down by about 24% with the great reduction of the broadcasting frequency of about 90%. These results demonstrated that the threshold had a greater impact on the collection delay than the total reward, and selecting a appropriate threshold was able to reduce the collection delay greatly at the expense of less total reward, which mitigated the collection delay and saved the system energy. Besides, as shown in Figure 6b and Figure 7b, with the increase of the number of available sensor nodes, the total reward rose and the collection delay decreased, both slightly. However, for the collection delay, the gap between the canonical Gittins index policy and our policy with σ=0.8 became wider with the number of sensor nodes increased, and conversely, the gap of the collection delay between the policies with σ=0.8 and σ=0.6 was narrowing. These two figures indicated that the number of sensor nodes had little effect on total reward and collection delay. Then, they also proved the effectiveness of the threshold in reducing the collection delay. After all, to balance the data collection quality and energy consumption better, the value of the threshold could be set based on the requirements of missions and the underwater environment during the application.

**Figure 8:**
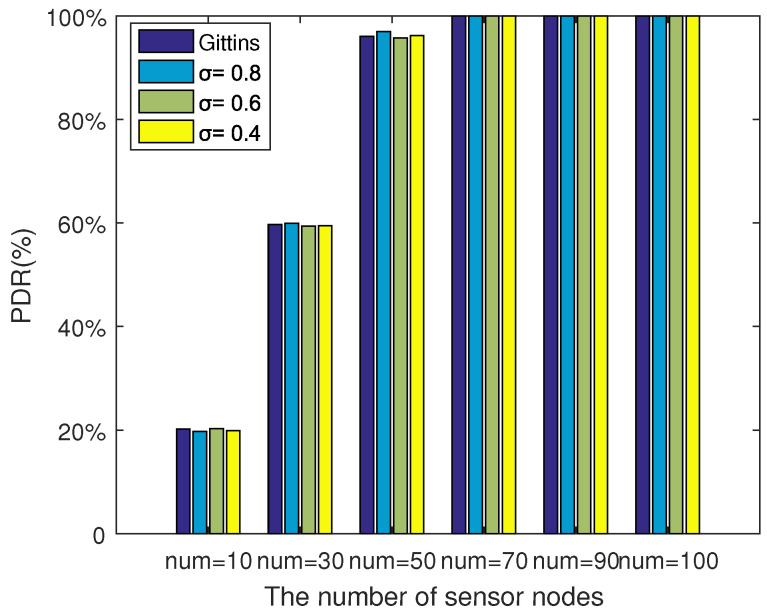
PDR comparison with different numbers of available sensor nodes.

Figure 8 shows the package delivery ratio (PDR) of the proposed policy with different thresholds as the sensor nodes increased. The PDR was defined as the ratio of the successful collected data packages to the total data packages sent by sensor nodes. We ran the simulation for 1000 time slots, repeated it 50 times, and took the average. This demonstrated that the PDR had little correlation with the threshold, but with the number of available sensor nodes. The more nodes, the higher the PDR would be, and when the number of sensor nodes climbed to 70, the PDR reached 100%. Therefore, a proper deployment of the underwater sensor network could optimize the performance to some extent.

## 6. Conclusions

In this paper, we fully considered the movement characteristics of the glider and proposed an indexable threshold policy for optimally scheduling the available sensor nodes in the communication range of the glider. We formulated the data collection process as an MAB problem and solved it by a modified Gittins index policy on which a threshold was set up to decrease the communication overhead and save the system energy. The whole data collection process was divided into the off-line part that calculated the index table offline and the on-line scheduling part that sensor nodes looked up the index table and acquired the index corresponding to its current state, which significantly reduced the computation complexity. Simulation results illustrated that the proposed sensor scheduling algorithm could effectively optimize the performance of data collection and maximize the network lifetime. Besides, the threshold could be flexibly set according to the mission requirements for a longer network lifetime with less BER loss, which was proven by the simulations. We addressed a feasible algorithm for the application of the glider to collecting sensor data from the UASN, and this topic deserves more research.

## Figures and Tables

**Table 1:** Notations.

Variables	Description	Variables	Description
*N*	Number of sensor nodes	$\varphi^{i}$	Transition probability of small-scale fading for one sensor node
*T*	Total time slots	$\Psi^{i}$	Transition probability matrix of small-scale fading for one sensor node
$B_{t}^{i}$	Channel state of one sensor node at one time slot	$\phi^{i}$	Transition probability of large-scale fading for one sensor node
$\zeta^{i}$	Small-scale fading state of one sensor node	$\Phi^{i}$	Transition probability matrix of large-scale fading for one sensor node
$\delta^{i}$	Large-scale fading state of one sensor node	$\theta^{i}$	Transition probability of residual energy for one sensor node
$x_{t}^{i}$	Current state of sensor node *i* at time slot *t*	$\Theta^{i}$	Transition probability matrix of residual energy for one sensor node
C	State space of small-scale fading	$p^{i}$	Transition probability for one sensor node
D	State space of large-scale fading	$P^{i}$	Transition probability matrix for one sensor node
E	State space of residual energy	$r^{i}$	System reward of one sensor node at one time slot
X	State space for one sensor	*W*	Energy consumption in one transmission

**Table 2:** Simulation parameters.

Parameter	Value
Glider Velocity	0.6 knots
Glider Depth Rating	0–1500 m
Glider Range	>500 km
Glider Noise	<6500 Hz
Number of Sensor Nodes	10–100
Node Initial Energy (e2)	100 J
Data Packet Size	1024 bits
Index Length	8 bits
Signal Frequency	30 kHz
